# Binding of Pro-Inflammatory Proteins S100A8 or S100A9 to Amyloid-β Peptide Suppresses Its Fibrillation

**DOI:** 10.3390/biom15030431

**Published:** 2025-03-17

**Authors:** Ekaterina A. Litus, Marina P. Shevelyova, Alisa A. Vologzhannikova, Evgenia I. Deryusheva, Andrey V. Machulin, Ekaterina L. Nemashkalova, Maria E. Permyakova, Andrey S. Sokolov, Valeria D. Alikova, Vladimir N. Uversky, Sergei E. Permyakov

**Affiliations:** 1 Institute for Biological Instrumentation, Pushchino Scientific Center for Biological Research of the Russian Academy of Sciences, Institutskaya Str., 7, Pushchino, 142290 Moscow, Russia; marina.shevelyova@gmail.com (M.P.S.); lisiks.av@gmail.com (A.A.V.); janed1986@ya.ru (E.I.D.); elnemashkalova@gmail.com (E.L.N.); mperm1977@gmail.com (M.E.P.); 212sok@gmail.com (A.S.S.); alikovalera@mail.ru (V.D.A.); permyakov.s@gmail.com (S.E.P.); 2 Skryabin Institute of Biochemistry and Physiology of Microorganisms, Pushchino Scientific Center for Biological Research of the Russian Academy of Sciences, pr. Nauki, 5, Pushchino, 142290 Moscow, Russia; and.machul@gmail.com; 3 Department of Molecular Medicine and USF Health Byrd Alzheimer’s Research Institute, Morsani College of Medicine, University of South Florida, Tampa, FL 33612, USA

**Keywords:** Alzheimer’s disease, amyloid fibrils, amyloid-β peptide, human serum albumin, S100 proteins, S100A8, S100A9, protein–protein interactions

## Abstract

Human serum albumin (HSA) is a natural depot of amyloid-β peptide (Aβ), a key player in Alzheimer’s disease (AD). HSA and pro-inflammatory Ca^2+^-binding proteins S100A8 and S100A9 are involved in Aβ metabolism and its deposition in the brain, serving as probable triggers and therapeutic targets in AD, but their interplay with regard to Aβ binding/fibrillation is unclear. To this end, here we explore the in vitro binding of Ca^2+^-bound S100A8 or S100A9 to monomeric Aβ and the influence of the S100 proteins on Aβ fibrillation. The equilibrium dissociation constants of the complexes of dimeric S100A8/S100A9 with Aβ40/42 estimated by biolayer interferometry are 1–5 µM. S100A8 and S100A9 interfere with HSA binding to Aβ. Thioflavin T assay and electron microscopy data show that micromolar S100A8/S100A9 inhibit Aβ40 fibrillation, and the inhibitory effect of S100A8 exceeds that for HSA. The competition for Aβ between HSA and S100A8/S100A9 may contribute to the Aβ-HSA imbalance in the pro-inflammatory conditions in AD.

## 1. Introduction

Alzheimer’s disease (AD) is a common multifactorial disease that is accompanied by steadily increasing cognitive and memory impairment, leading to dementia [1,2]. The main pathomorphologic hallmarks of AD are extracellular senile plaques (deposits of amyloid-β peptide, Aβ, with other components) and intracellular accumulations of the abnormally modified tau protein associated with the progressive atrophy of cortical and subcortical structures [2,3]. AD is subdivided into familial and sporadic forms, with the latter accounting for about 90% of all AD cases [4]. The familial form of AD is associated with mutations in the genes for the amyloid precursor protein (APP) and the proteins involved in its processing, Presenilin-1 and Presenilin-2 [3,4]. The etiology of sporadic and typically late-onset AD is less understood and is associated with a combination of rare genetic variants and environmental factors [4]. Infections, inflammation, oxidative stress, and the impaired activity of neurotransmitters favor the development of sporadic AD along with the accumulation of neurotoxic Aβ oligomers and neurofibrillary tangles [5,6,7,8]. Meanwhile, it is disturbances in the metabolism of tau protein and Aβ that are considered to be the defining components of the pathogenesis of sporadic AD [2].

The imbalance between Aβ synthesis and its excretion from the central nervous system (CNS) in AD leads to Aβ accumulation in brain tissue [9]. Elevated levels of neurotoxic Aβ oligomers cause neuronal cell death with the subsequent development of cognitive impairment [10]. The same effect is achieved with improper posttranslational modifications of tau protein [11,12]. The debate as to which of these interrelated mechanisms is crucial for triggering and progression of AD is still ongoing [13,14,15,16]. In favor of the key role of amyloids is the fact that AD does not develop in the absence of amyloid deposits, and genetic forms of only Aβ are associated with AD. The recent approval of the Aβ-specific monoclonal antibodies for the treatment of mild cognitive impairment and mild AD (aducanumab, lecanemab, donanemab [17]) is consistent with this conclusion.

It is important to note that the Aβ pool is heterogeneous. Aβ with an amino acid chain length of 40 residues (Aβ40) is the most abundant and accounts for more than 50% of the Aβ pool in cerebrospinal fluid (CSF) [18,19]. The other major isoform is Aβ42, whose content is ~5–10% [18,19] and which has a tendency toward oligomerization and increased cytotoxicity [20,21]. Aβ42 is found in all amyloid deposits, whereas Aβ40 is detected in ~30% of amyloid deposits and is associated with mature plaques [22]. Moreover, a reduced Aβ42/Aβ40 ratio in blood plasma is an important marker of Aβ accumulation in the brain [23].

In addition to Aβ isoforms, senile plaques contain metal ions (Cu, Fe, Zn) and dozens of proteins, including immunoglobulins, components of the complement system, and proteins involved in metabolism, molecular transport, blood coagulation/hemostasis, cell adhesion, the extracellular matrix, etc. [24,25,26,27]. Among them, human serum albumin (HSA) is of particular importance, since this major protein of serum and CSF serves as a depot of Aβ [28,29] due to its ability to bind monomeric and multimeric forms of Aβ and also prevents its fibrillation [29,30,31,32]. The latter fact indicates the possibility that Aβ pathology develops specifically in the extracellular space of the brain due to reduced HSA levels in the CSF compared to that in the blood [29]. The intracerebroventricular infusion of HSA in the 3xTg mouse model provided multifactorial beneficial effects, leading to improvement in cognitive tests [33]. Plasma Aβ clearance by plasma exchange and the replacement of endogenous albumin with therapeutic albumin slowed cognitive and functional decline in clinical trials in patients with mild-to-moderate AD [34,35].

Another component colocalized with senile plaques and associated with AD are specific members of the S100 protein family (reviewed in ref. [36]). The S100 family contains over two dozen small (9–13 kDa) multifunctional Ca^2+^-binding proteins of the EF-hand superfamily [37,38,39,40], defined by the presence of the EF-hand motif, which coordinates a calcium ion through a loop located between two α-helices [41,42]. Some S100 proteins affect cognitive processes, neuronal development and maintenance, APP processing, Aβ levels, the formation of amyloid plaques, post-translational modifications of tau protein, trace metal homeostasis, and signaling pathways related to AD progression [36]. The AD-related S100 proteins include S100A1, S100A6, S100A7, S100A8, S100A9, S100A12, and S100B [36]. With the exception of S100A7, they are found near or within amyloid plaques in the brains of AD animal models or AD patients [26,27,36,43,44,45]. Of these, S100A8 and S100A9 stand out as proinflammatory proteins contributing to the neuroinflammation that accompanies AD progression [36,46,47,48,49,50,51]. In mouse AD models, S100A8 and S100A9 accumulate in microglia cells surrounding amyloid plaques [49], and S100A8 is deposited in the center of amyloid plaques [26]. Similarly, S100A9 is included in the amyloid plaques of AD patients [27]. S100A9 is inherently amyloidogenic and synergistically promotes Aβ40 fibrillation in vitro [43,52], which is partly due to direct S100A9–Aβ40 interaction, as evidenced by nuclear magnetic resonance (NMR) spectroscopy [43]. S100A9 plaques are found in the brains of patients with traumatic brain injury [43,53], which is considered a risk factor for AD [54]. Similarly, studies in mice AD models have revealed the formation of extracellular S100A8 aggregates prior to the accumulation of Aβ plaques and a feedback loop between S100A8 and Aβ production [55]. In contrast to the effect of S100A9 on Aβ40 fibrillation [43,52], S100A8 suppresses the fibrillation of Aβ40 and Aβ42 in vitro [26,56]. S100A9 levels in CSF have been proposed as a marker of the early stages of cognitive impairment in AD [57]. In addition, the deficiency of S100A9 reduced amyloid plaque burden and prevented cognition decline in a mouse AD model [49,50].

Although S100A8, S100A9, and Aβ’s natural depot, HSA, serve as possible triggering factors and therapeutic targets in AD, their interplay with respect to Aβ binding/fibrillation remains unclear. It should be mentioned that the study of monomeric Aβ is particularly important, since interactions of ligands with such a state of Aβ can modulate the process of its fibril formation. The search for the strong binders of protein monomers is one of the strategies for finding therapeutically significant inhibitors of fibrillation, including Aβ [58]. We previously evaluated HSA affinity for monomeric isoforms of Aβ [30,59]. In the present study, we aimed at the quantitation of the interaction of Ca^2+^-loaded forms of S100A8 and S100A9 with monomeric Aβ and the examination of the effect of the S100 proteins on Aβ fibrillation compared with HSA.

## 2. Materials and Methods

### 2.1. Materials

Albumin, non-denatured human serum [60] (cat.#126654), and anti-amyloid-β (oligomer) antibody, clone F11G3 (MABN1839), were from Merck (Rahway, NJ USA). Recombinant human Aβ40/Aβ42 (Aβ40 or Aβ42) was prepared in *E. coli* as described earlier [61]. Briefly, after cell disruption, 6–His–ubiquitin–Aβ fusion protein was purified by immobilized metal affinity chromatography (IMAC, Bio-Rad Laboratories (Hercules, Clearwater, FL, USA)). Further, after the proteolysis of fusion protein using the catalytic core of ubiquitin carboxyl-terminal hydrolase 2 (Usp2-cc), Aβ40/Aβ42 was finally purified by sequential IMAC and reversed-phase chromatography on a Jupiter C18-column (Phenomenex^®^). The purity and homogeneity of the obtained Aβ40/Aβ42 preparations were determined by electrophoresis in polyacrylamide gel (PAAG) and mass spectrometry. Recombinant human S100A8/S100A9 (S100A8 or S100A9) was prepared in *E. coli* as described in our previous paper [62]. Briefly, after cell disruption, the ubiquitin–S100A8 chimera was purified using a Profinity IMAC column. After proteolysis with Usp2-cc peptidase, S100A8 protein was loaded onto a TOYOPEARL^®^ SuperQ-650M column (Tosoh Bioscience (Tokyo, Japan)) and eluted by a linear gradient of NaCl. The collected S100A8 protein was further purified using a HiPrep—26/60 Sephacryl^®^ S-100 HR column (GE Healthcare (Chicago, IL, USA)). S100A9 was purified by the centrifugation of disrupted cells followed by the incubation of the supernatant with 50% ammonium sulfate. The supernatant was dialyzed and loaded onto a TOYOPEARL^®^ SuperQ-650M column. S100A9 protein was eluted by a linear gradient of NaCl followed by chromatography on SP Sepharose^®^ Fast Flow and then HiPrep—26/60 Sephacryl^®^ S-100 HR columns (both from GE Healthcare (Chicago, IL, USA)). The purified S100A8/S100A9 proteins were dialyzed and stored in 10% glycerol at −70 °C. Usp2-cc was expressed in *E. coli* and purified mainly in accordance with ref. [63].

Ultra-grade Tris and Tricine, ethylenediaminetetraacetic acid (EDTA), dimethyl sulfoxide (DMSO), and isopropyl β-D-1-thiogalactopyranoside (IPTG) were from Helicon (Moscow, Russia). 2-Mercaptoethanol (2-ME) was from VWR Life Science (Solon, OH, USA). Urea, glycerol, and ampicillin were from NeoFroxx (Einhausen, Germany). Sodium chloride, potassium chloride, Coomassie Brilliant Blue R-250, and sodium azide were from Dia-M (Moscow, Russia). Imidazole, sodium hydroxide, sodium dodecyl sulfate (SDS), and DL-dithiothreitol (DTT) were purchased from Panreac AppliChem (Darmstadt, Germany). Calcium/magnesium chloride were from Fluka (Charlotte, NC, USA). 1-Ethyl-3-(3-dimethylaminopropyl)carbodiimide (EDAC), N-hydroxysuccinimide (sulfo-NHS), thioflavin T (ThT), acetonitrile, and polyethylene glycol sorbitan monolaurate (TWEEN^®^) 20 were from Sigma-Aldrich (St. Louis, MO, USA). Ethanolamine was purchased from Bio-Rad Laboratories (Hercules, USA). Hydrochloric acid was from Sigma Tec LLC. (Moscow, Russia). Phenylmethylsulfonyl fluoride (PMSF) was from Amresco^®^ LLC. (Vienna, Austria). Trifluoroacetic acid (TFA) was purchased from Fisher Scientific (Madrid, Spain). Acetic acid and ammonium hydroxide were from Chimmed (Moscow, Russia) and Component-reaktiv (Moscow, Russia), respectively.

Protein concentrations were measured spectrophotometrically using molar extinction coefficients at 280 nm calculated according to ref. [64]: 34,445 M^−1^cm^−1^ for HSA, 11,460 M^−1^cm^−1^ for S100A8, 6990 M^−1^cm^−1^ for S100A9, 41,370 M^−1^cm^−1^ for Usp2-cc and 1490 M^−1^cm^−1^ for Aβ40/Aβ42 at pH 7.4–8.0. Stock solution of ThT (0.8 mg/mL) was prepared in distilled, deionized water. The ThT concentration was measured spectrophotometrically using the molar extinction coefficient at 412 nm of 36,000 M^−1^cm^−1^ [65].

### 2.2. Bio-Layer Interferometry Studies

BLI measurements of S100A8/S100A9/HSA affinity to monomeric Aβ40/Aβ42 samples at 25 °C were performed using a ForteBio Octet^®^ QKe System (Fremont, CA, USA) with Aβ immobilization on the sensor surface. Next, 96-well microplates with analyte solutions were kept at 25 °C and shaken at 1000 rpm. The Aβ samples were pretreated with neat TFA, followed by dissolution in DMSO as described in ref. [61,66]. The pretreated ligand (5 μg/mL Aβ40/Aβ42 in 10 mM sodium acetate, pH 4.5 buffer) was immobilized onto Octet^®^ AR2G biosensor (Sartorius AG, Göttingen, Germany) by amine coupling using EDAC/sulfo-NHS to achieve the loading level of 3.5 nm. The rest of the activated amine groups on the sensor were blocked by 1 M ethanolamine solution. The noncovalently bound ligand molecules were washed off the sensor with a 0.5% SDS water solution, followed by passage of the assay buffer (20 mM Tris-HCl, 140 mM NaCl, 4.9 mM KCl, 2.5 mM CaCl_2_, 1 mM MgCl_2_, pH 7.4), resulting in the loading level of 1.5 nm. The absence of Aβ oligomers has been confirmed by the use of the Aβ oligomer-specific antibodies as an analyte (10–100 nM), which did not reveal a noticeable BLI response. The baseline was recorded for 120 s, followed by association with the analyte (30–50 μM S100A8/S100A9; 30–50 μM HSA; 67 μM HSA in the presence/absence of 12 µM S100A8 or 7 µM S100A9) for 600 s and the dissociation of the complex for 600/1200 s. The sensor surface was regenerated by the passage of 0.5% SDS water solution for 30 s.

The response data were corrected for non-specific binding by subtracting the signal from the reference sensors (one lacking the immobilized analyte (A) in the presence of ligand (L) and one with the analyte in the absence of the ligand in the buffer. The resulting BLI sensograms were analyzed using either a bivalent analyte model:

(1) A+Lka1⇄kd1AL     AL+Lka2⇄kd2ALL      KD1                        KD2          

or a heterogeneous ligand model:

(2) A+L1ka1⇄kd1AL1     A+L2ka2⇄kd2AL2        KD1                        KD2          

where *k_a_* and *k_d_* are kinetic association and dissociation constants, respectively, and *K_D_* represents equilibrium dissociation constants. The constants were evaluated for each analyte concentration using ForteBio Data Analysis software v.12.0 (Fremont, CA, USA), followed by averaging the resulting values (n = 3–4; standard deviations are indicated).

### 2.3. ThT Fluorescence Assay

The human Aβ40 samples were pretreated as described in ref. [61] with some modifications. Aβ40 sample was dissolved in 5 mM NaOH at pH 11.8 (0.5 mg/mL) and then rocked gently for 72 h at 4 °C. ThT fluorescence emission was measured mainly as described in ref. [61]. Aβ40 at 20 µM in 25 mM Tris-HCl, 140 mM NaCl, 4.9 mM KCl, 2.5 mM CaCl_2_, 1 mM MgCl_2_, and pH 7.4 buffer with 0.05% NaN_3_ was incubated with 10 µM ThT in the absence/presence of 5 µM HSA, 4 µM S100A8 or 4 µM S100A9 at 30 °C. The ThT fluorescence intensity (λ_em_ = 485 nm, λ_exc_ = 440 nm) was recorded every 30 min for 266 h using a BioTek Synergy H1 multimode microplate reader (Agilent Technologies, Inc., Santa Clara, CA, USA) with orbital shaking prior to each measurement. Each measurement was performed in 3–4 repetitions. The control kinetic curves (for the wells without Aβ40) were subtracted from the corresponding kinetic curves of the experimental samples. The control kinetic curves are shown in the Supplementary Materials (Figure S4). Data are presented as the mean ± standard deviation. The effect of S100A8 (4 µM), S100A9 (4 µM), and HSA (5 µM) on ThT fluorescence in samples of mature Aβ fibrils was additionally monitored. The corresponding kinetic curves are given in the Supplementary Materials (Figures S7 and S8).

### 2.4. Transmission Electron Microscopy

A 300-mesh copper grid coated with a 0.2% formvar film was placed onto a 10 µL drop of the sample. After allowing the sample (after the ThT fluorescence assay) to adsorb for 10 min, the grid was negatively stained for 2 min using a 1% (*w*/*v*) aqueous solution of uranyl acetate. Excess staining solution was removed with filter paper, followed by a 1 min rinse in deionized water. The samples were examined using a JEM-1400Plus (HC) transmission electron microscope (JEOL, Ltd., Tokyo, Japan) operated at 80 keV.

### 2.5. Structural Modeling of Aβ40-S100 Complexes

The molecular modeling was based on the structures of human Aβ40 and Ca^2+^-loaded dimers of human S100A8 and S100A9: PDB [67] entries 2LFM (NMR, model 1), 1MR8 (X-ray, chains A, B), and 5I8N (NMR, model 1, chains A, B, mutation C3S), respectively. The models of the tertiary structures of Aβ40-S100A8/S100A9 complexes were built using the ClusPro docking server (accessed on 1 July 2024) [68]. The analysis of the distributions of the predicted contact residues of the proteins over their amino acid sequences within the models was performed as described in [69]. Ten representative models of Aβ40-S100A8/S100A9 complexes were overlayed. The numbering of the contact residues is according to the PDB entries. The model tertiary structures were drawn using the molecular visualization system PyMOL v.2 [70] (accessed on 1 July 2024).

### 2.6. Dynamic Light Scattering Measurements

Dynamic light scattering (DLS) measurements were carried out using a Zetasizer Nano ZS system (Malvern Instruments Ltd., Malvern, UK). The backscattered light from a 4 mW He-Ne laser 632.8 nm was collected at an angle of 173°. S100A8 (4.0, 6.8, 13.6, 27.3 and 54.5 µM) and S100A9 (3.5, 7.1, 14.2, 28.4, 56.8 µM) solutions in 20 mM Tris-HCl, 140 mM NaCl, 4.9 mM KCl, 2.5 mM CaCl_2_, 1 mM MgCl_2_, and pH 7.4 buffer were kept at 25 °C. The acquisition time for a single autocorrelation function was 100 s. The resulting autocorrelation functions are averaged values from three measurements. The volume-weighted size distributions were calculated using the following parameters for the buffer: a refractive index of 1.334 measured with an RL3 refractometer (PZO, Warszawa, Poland) and the viscosity value *η* = 0.95 mPa·s measured using a micro-rheology method with a water suspension of standard latex nanoparticles. Molecular mass corresponding to the volume-weighted hydrodynamic radius was calculated according to the equations from [71] in approximation of a globular protein.

### 2.7. Chemical Cross-Linking of Proteins

The cross-linking of S100A8 (25, 50, and 75 μM), 20 μM Aβ40/Aβ42, and their mixtures with 0.02% glutaraldehyde was performed in 20 mM HEPES-KOH, 140 mM NaCl, 4.9 mM KCl, 2.5 mM CaCl_2_, 1 mM MgCl_2_, pH 7.4 buffer at 37 °C for 1 h, mainly as described in ref. [72]. The protein solution was incubated at 25 °C for 30 min prior to the cross-linking. The samples were subjected to Tricine-SDS-PAGE under reducing conditions (5% concentrating and 15% resolving gels) and staining with Coomassie Brilliant Blue R-250.

## 3. Results

### 3.1. The Interaction of S100A8/S100A9 Proteins with Aβ Peptides

The direct interaction of S100 proteins with monomeric Aβ was studied by biolayer interferometry in the buffer conditions mimicking the physiological conditions (20 mM Tris-HCl, 140 mM NaCl, 4.9 mM KCl, 2.5 mM CaCl_2_, 1 mM MgCl_2_, pH 7.4). Recombinant Aβ40/Aβ42 was immobilized on the surface of the BLI sensor by amine coupling, followed by washing the sensor with a 0.5% aqueous SDS solution to ensure the monomeric state of Aβ (the latter was confirmed using the antibodies specific to Aβ oligomers). It should be noted that the fixation of Aβ in the monomeric state allows us to avoid the coexistence in the reaction mixture of a set of oligomeric forms, which would be significantly different for Aβ40 and Aβ42 [21]. It increases the reliability of the results obtained and allows us to compare the parameters of the formation of complexes between S100A8/S100A9 proteins and Aβ40/Aβ42.

**Figure 1:**
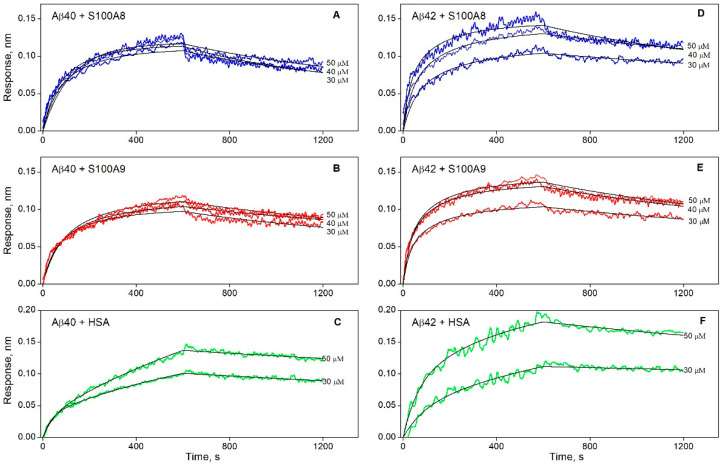
Kinetics of interaction between monomeric Aβ40 (panels **A**–**C**) or Aβ42 (**D**–**F**) and S100A8 (blue), S100A9 (red), or HSA (green) at 25 °C, monitored using BLI (20 mM Tris-HCl, 140 mM NaCl, 4.9 mM KCl, 2.5 mM CaCl_2_, 1 mM MgCl_2_, pH 7.4). The analyte (S100/HSA) concentrations are indicated near the sensograms. The black curves are theoretical, calculated according to either the bivalent analyte (1) (panels **A**,**B**,**D**,**E**) or the heterogeneous ligand (2) (**C**,**F**) models (see Table 1 and Table 2 for the fitting parameters).

The BLI sensograms for Ca^2+^-bound forms of S100A8/S100A9 proteins (30–40 μM) exhibited well-defined concentration-dependent effects, reflecting both association and dissociation phases (Figure 1). Taking into consideration that S100A8/S100A9 are prone to dimerization/tetramerization in water solutions [73], their average degrees of multimerization were estimated using DLS: 2.20 ± 0.27 and 2.54 ± 0.30 for S100A8 and S100A9, respectively (protein concentration of 4–57 μM). Since the S100 proteins are predominantly dimeric, their BLI sensograms were described within the bivalent analyte model (1), taking into account that the dimer concentration is half of the monomer concentration. Considering that the kinetic and equilibrium parameters of the S100–Aβ interactions estimated for each BLI sensogram did not reveal dependence on the analyte concentration, their resulting estimates were averaged (Table 1).

S100A8 and S100A9 exhibit nearly identical equilibrium and kinetic parameters of interaction with Aβ42 (Table 1). Meanwhile, the affinity of S100A9 for Aβ40 is about 2-fold higher than that of S100A8, mainly due to the slower dissociation of the S100A9–Aβ40 complex. The affinity of S100A8 for Aβ42 is 4-fold higher than that for Aβ40, due to 1.5-fold accelerated association and 2.4-fold slower dissociation. Notably, our estimates of the equilibrium dissociation constants (*K_D_*) for S100A8/S100A9 complexes with Aβ40/Aβ42 (1–5 μM) are close to some values reported for other targets of S100A8/S100A9: S100A9 binds the RAGE V domain with *K_D_* of 5–6 μM [74] and S100A12 with *K_D_* of 1.3 μM [75], S100A8 binds Toll-like receptor 4 with *K_D_* of 1 μM in the presence of Ca^2+^ and Zn^2+^ [76]. This fact indicates the physiological relevance of S100–Aβ interactions.

The interactions of S100A8 [26] and S100A9 [43,52] proteins with Aβ have been previously reported, but they were not quantified and likely correspond to multimeric forms of Aβ due to the lack of means of its monomerization. Instead, these studies demonstrate the influence of S100A8/S100A9 on the process of Aβ fibrillization. In fact, even relatively low levels of Aβ (20 μM) are accompanied by the accumulation of its multimeric states, as evidenced by chemical cross-linking data (Figure S1). Nevertheless, Aβ multimerization does not preclude its interaction with S100A8, and the complex of their monomers is detected by chemical cross-linking, as evidenced by the appearance of the faint band at about 15 kDa (Figure S1).

### 3.2. Modeling of Aβ40-S100A8/S100A9 Complexes

**Figure 2:**
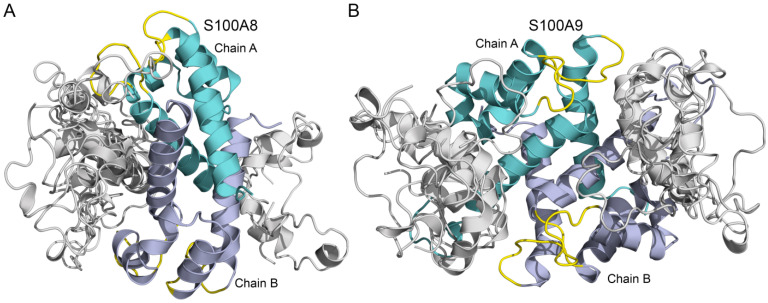
Overlay of the 10 representative models of S100A8–Aβ40 (**A**) and S100A9–Aβ40 (**B**) complexes calculated using the ClusPro docking server [68]. Aβ40 monomers are shown in grey. Chains A and B of S100A8/S100A9 dimers are shown in cyan and dark grey, respectively; their Ca^2+^-binding loops are highlighted in yellow.

The tertiary structures of the complexes between the Ca^2+^-loaded dimers of S100A8/S100A9 and Aβ40 monomer were built using the ClusPro docking server [68] (Figure 2). The overlay of 10 representative structures predicted for the S100A8–Aβ40 complex (Figure 2A) shows that Aβ40 preferentially binds to the groove between chains A and B of S100A8 (Figure 2A), which is considered a typical binding site of S100 proteins for numerous protein targets, including cytokines and receptors [77,78,79]. The predicted contact residues for chain A of S100A8 include I60 (from the loop between helices α3 and α4), Q69, I73, and K77 (helix α4). The contact residues of chain B include K56 (helix α3), I60 (the loop between helices α3 and α4), I73, I76, and K77 (helix α4). The analogous modeling of the S100A9–Aβ40 complex (Figure 2B) predicts that chain A of S100A9 binds Aβ40 via residues K51 and H61 (helix α3), R85, W88, A89 and E92 (helix α4), and H103 and H105 (C-terminus). The predicted contact residues of chain B are nearly the same: K51, R85, W88, E92, H103, and H105. Our results are consistent with the modeling of the S100A9–Aβ40 complex reported earlier [43], which showed that residues R85 and L86 make particularly close contacts with Aβ40. The residue S26 of Aβ40 is predicted to bind S100A9, which is consistent with the literature data [43]. The modeling results predict specific binding that may explain the inhibition of amyloid formation. Namely, different non-complementary amino acid sequences of S100A8/S100A9 and Aβ40 could be integrated into the same β-sheet cross-link during the binding process. The obtained data can be used in the future to modulate the enhancement of the interaction of S100A8/S100A9 with Aβ40.

### 3.3. S100A8/S100A9 Prevent HSA Interaction with Monomeric Aβ

Since HSA serves as a natural depot of Aβ [28,29], an intriguing question is whether S100A8/S100A9 can affect Aβ binding to HSA. The BLI sensograms for HSA (30–50 µM) interaction with monomeric Aβ40/Aβ42 immobilized on a BLI sensor are described by the *heterogeneous ligand* model (2) (Figure 1C,F). The resulting *K_D_* values (Table 2) are in agreement with our previous surface plasmon resonance (SPR) estimates [30,59]. 

**Figure 3:**
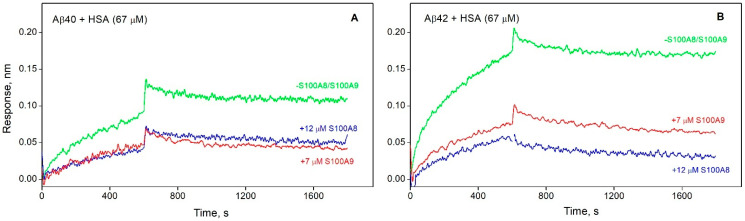
Kinetics of HSA (67 µM) interaction with monomeric Aβ40 (**A**) or Aβ42 (**B**) immobilized on a BLI sensor in the absence (green) or presence of S100A8 (12 µM, blue) or S100A9 (7 µM, red) at 25 °C, monitored using BLI (20 mM Tris-HCl, 140 mM NaCl, 4.9 mM KCl, 2.5 mM CaCl_2_, 1 mM MgCl_2_, pH 7.4).

Notably, the HSA affinity for monomeric Aβ exceeds that for S100A8/S100A9 (Table 1): the *K_D_* values for the Aβ40-S100A8 or Aβ40-S100A9 complexes are, respectively, 23- or 10-fold higher than the lowest *K_D_* value for the HSA–Aβ40 interaction, whereas for Aβ42, this effect is much less pronounced (3-fold difference). Meanwhile, S100A8/S100A9 notably suppress HSA interaction with Aβ40/Aβ42: a similar experiment performed in the presence of 12 µM S100A8 or 7 µM S100A9 shows a marked decline in the maximal BLI signal (Figure 3). The observed prevention of HSA interaction with monomeric Aβ in the presence of S100A8/S100A9 could be due in part to direct S100 binding to HSA, but this possibility was ruled out by control BLI experiments, which did not show S100–HSA interaction (Figure S2). Therefore, this effect is mainly due to competition between HSA and S100A8/S100A9 for Aβ molecules.

The fact that S100A8, S100A9, and HSA are found in amyloid deposits in the brain tissue of AD patients [26,43,80] suggests their involvement in the Aβ intermolecular interactions and the subsequent Aβ fibrillation. Our findings evidence that S100A8 and S100A9 proteins preclude HSA interaction with Aβ through a competitive mechanism. The latter suggests that HSA loading with Aβ will depend on the relative concentrations of HSA, S100A8/S100A9, and Aβ. Total Aβ concentrations in CSF and blood serum do not exceed nanomolar levels [19,81,82,83]. Meanwhile, concentrations of HSA and S100A8/S100A9 greatly depend on the fluid: HSA concentration ranges from 3 μM in CSF to 645 μM in serum [84], while the total concentration of S100A8 and S100A9 varies from 0.09 μM in the serum of a healthy donor [85] to 3–6 μM in the synovial fluid during inflammation [86]. Furthermore, albumin synthesis and secretion by microglial cells is enhanced upon their activation by Aβ42 [87]. In addition, local brain concentrations of Aβ and S100A8/S100A9 in the sites of amyloid deposition may be quite high [88]. Our theoretical estimates indicate that at the Aβ and HSA concentrations equivalent to those in CSF [19,83,89,90] and the total S100A8 and S100A9 contents corresponding to those in the inflammatory focus [86], 12% of Aβ40 and 36% of Aβ42 will be bound to S100A8 and S100A9, while 82% of Aβ40 and 56% of Aβ42 will be bound to HSA (Appendix A). Therefore, S100A9 and S100A8 should be considered possible competitors of HSA with respect to Aβ binding, with S100A9 being more efficient than S100A8.

### 3.4. Effect of S100A8/S100A9 on Aβ Fibrillation In Vitro

**Figure 4:**
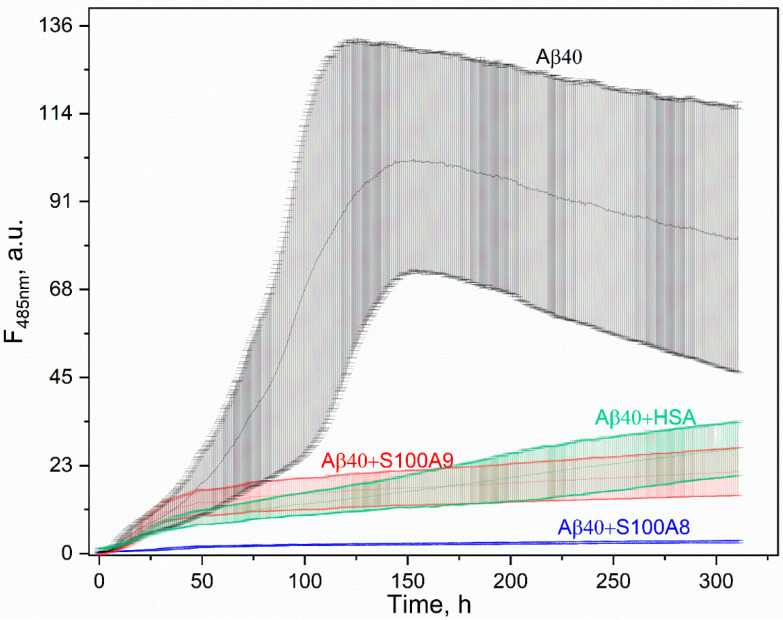
Kinetics of Aβ40 (20 μM) fibrillation in the presence of HSA (5 μM), S100A8 (4 μM), or S100A9 (4 μM) at 30 °C, followed by ThT (10 μM) fluorescence intensity at 485 nm (25 mM Tris-HCl, 140 mM NaCl, 4.9 mM KCl, 2.5 mM CaCl_2_, 1 mM MgCl_2_, pH 7.4 buffer with 0.05% NaN_3_). Excitation wavelength of 440 nm. The standard deviations of the fluorescence signals are indicated.

We investigated the effect of Ca^2+^-loaded S100A8/S100A9 on the kinetics of the Aβ40 fibrillation at 30 °C using thioflavin T fluorescence assay (Figure 4) and S100A8/S100A9 at a concentration of 4 μM, which corresponds to that in the inflammatory focus [86]. The addition of S100A8 dramatically decreased the maximum fluorescence signal (*I_max_*) by 32-fold compared to the sample containing Aβ40 alone. A similar but less pronounced effect is observed in the presence of S100A9 (*I_max_* is decreased by a factor of 5). HSA at a concentration of 5 μM (close to that in CSF [84]) lowers the *I_max_* value by 4-fold, which is consistent with previous reports [29,61]. This effect is close to that for S100A9 but lower than the effect observed for S100A8.

**Figure 5:**
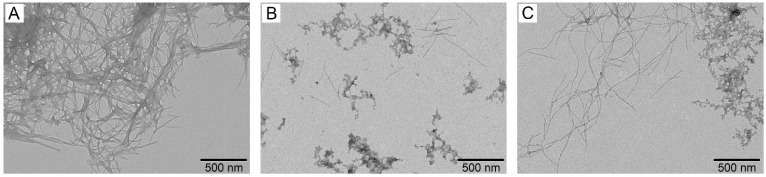
Negative staining TEM images of the 20 μM Aβ40 fibers grown in the course of the ThT fluorescence assay (**A**) and in the presence of 4 μM S100A8 (**B**) or 4 μM S100A9 (**C**). The scale bars represent 500 nm.

The data of the ThT fluorescence assay were confirmed by electron microscopy analysis (Figure 5). The incubation of the Aβ40 sample (20 μM) reveals dense clusters of the intertwined mature fibrils reaching microns in length (Figure 5A). The fibrillation in the presence of 4 μM S100A9 gives rise to drastically more fuzzy fibrils, which indicates the suppression of the process (Figure 5C). The Aβ40 fibrillation in the presence of 4 μM S100A8 yields even fewer fibrils (Figure 5B), in accord with the ThT fluorescence assay (Figure 4). No fibrils were detected in S100A8/S100A9 control samples without Aβ40 (Figures S5 and S6).

**Figure 6:**
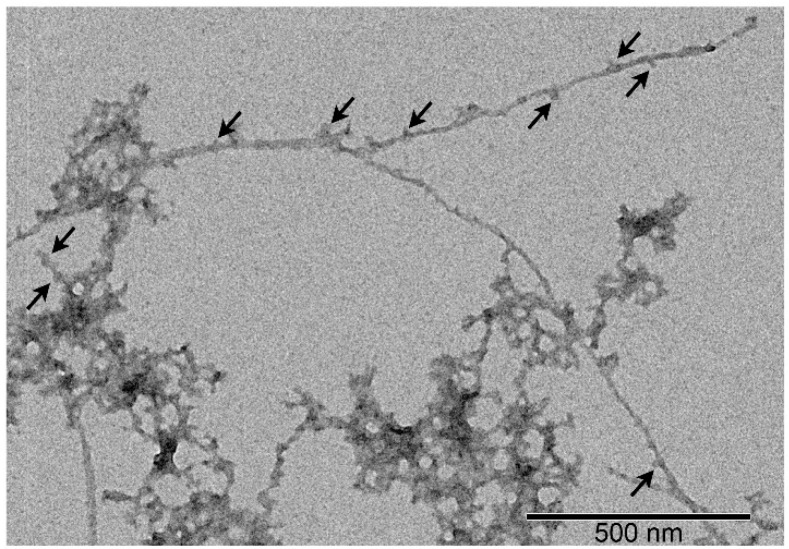
Negative staining TEM image of the complex between Aβ40 (20 μM) fibers and S100A9 (4 μM). The scale bar represents 500 nm. The arrows indicate the probable sites of S100A9 adhesion to Aβ40 fibrils.

S100A8 and S100A9 are known to be able to form aggregates and fibrils under certain conditions [91,92]. The previous AFM study of the interaction between Aβ42 and S100A9 fibrils suggested that the bulk of co-aggregated complexes is represented by Aβ42 amyloids templating S100A9 fibrils on their surfaces [92]. The TEM image for the analogous S100A9–Aβ40 complex (Figure 6) also suggests that S100A9 “sticks” to Aβ40 fibrils, consistent with the protein interaction mechanism described in ref. [92]. Taken together, the TEM data evidence a suppressive effect of S100A8/A9 on Aβ40 fibrillation, in accord with the ThT fluorescence assay data.

Although our results evidence that 4 μM S100A9 suppresses Aβ40 fibrillation (Figure 4 and Figure 5C), S100A9 at higher concentrations of 20–200 µM has previously been shown to exert the opposite effect [43,52]. This discrepancy may be due to differences in the experimental conditions, including S100A9 concentration, the temperature of the experiment (30 °C versus 37 °C), and the presence of Ca^2+^ and Mg^2+^ excess in our experiments to mimic the physiologically relevant conditions.

Our data and the literature data on Aβ40 fibrillation are in partial agreement with the effects of S100A9 and S100A8 on Aβ42 fibrillation reported earlier [26,92]. J. Pansieri et al. demonstrated that the lag phase of the Aβ42 fibrillation is more pronounced at the lowest S100A9 concentration of 2 μM, although the inhibitory effects persisted at S100A9 concentrations up to 100 μM [92]. According to the authors, S100A9 co-aggregates with Aβ42, affecting fibril morphology. Meanwhile, S100A8 at a concentration of 16 μM slows but does not completely inhibit the Aβ42 fibrillation [26].

Examination of the additivity of the effects of HSA (5 µM) and S100A8/S100A9 (4/2 µM) with regard to Aβ40 fibrillation showed that the addition of HSA does not cause qualitative changes in the individual effects of S100A8 and S100A9 proteins (Figure S3). Therefore, S100A9 and S1008 proteins at physiologically relevant concentrations prevent the formation of Aβ40 fibrils regardless of the micromolar level of HSA.

## 4. Conclusions

Accumulating evidence suggests the active involvement of the proinflammatory proteins S100A8 and S100A9 in Aβ metabolism in the CNS. Positive feedback between S100A8 and Aβ productions has previously been found, which is hypothesized to be critical for the accumulation of Aβ deposits [55]. Similarly, S100A9 favors APP processing and Aβ accumulation [49]. Aside from signaling through an array of cell surface receptors [49,55], S100A8 and S100A9 are capable of aggregation and co-deposition with Aβ, which was observed in AD patients and in mouse models of AD [26,43,91]. The primary Aβ depot, HSA, is also included in the amyloid deposits [26,43,80], but the interplay between Aβ, HSA, and S100A8/S100A9 proteins and its significance for amyloid formation remains obscure. In the present work, we have described the specific processes related to this issue, starting with a numerical characterization of the individual chemical equilibria occurring in the system and ending with a consideration of the complex phenomenon of Aβ fibrillation in the presence of the other reactants and their combinations. Despite the relatively low affinity of S100A8 and S100A9 homodimers for monomeric Aβ40/Aβ42 compared with HSA, the S100 proteins markedly suppress both HSA binding to Aβ40/Aβ42 monomer and Aβ40 fibrillation in vitro, with S100A8 most efficiently blocking the latter process. The competition between HSA and S100A8/S100A9 for Aβ likely contributes to the Aβ-HSA imbalance in the pro-inflammatory conditions in AD favoring elevated S100A8/S100A9 levels. In this way, these S100 proteins may link inflammation to the impaired Aβ metabolism in AD. Given that the accumulation of S100A8 aggregates precedes Aβ plaque formation in murine models of AD [55], exploiting its protective function or targeted prevention of S100A8 accumulation or its interaction with Aβ or its other targets may be a viable therapeutic strategy, as demonstrated for S100A9 in ref. [49,50]. Further in-depth in vivo studies are needed to choose an effective approach and validate it experimentally.

## Figures and Tables

**Table 1:** Parameters of the bivalent analyte model (1) describing the BLI data on the kinetics of the S100 interactions with monomeric Aβ shown in Figure 1.

	**K_D_ × 10^6^, M**	k_a_ × 10^−2^, M^−1^s^−1^	k_d_ × 10^4^, s^−1^
Aβ40			
S100A8	**5.1 ± 2.3**	2.2 ± 0.7	10.9 ± 1.5
S100A9	**2.1 ± 1.3**	2.6 ± 1.0	5.4 ± 1.0
Aβ42			
S100A8	**1.3 ± 0.9**	3.4 ± 0.9	4.6 ± 1.9
S100A9	**1.2 ± 0.6**	4.5 ± 1.3	5.1 ± 1.1

**Table 2:** Parameters of the heterogeneous ligand model (2) describing the BLI data on the kinetics of the HSA–Aβ interaction shown in Figure 1C,F.

**K_D1_ × 10^6^, M**	k_a1_ × 10^−2^, M^−1^s^−1^	k_d1_ × 10^4^, s^−1^	**K_D2_ × 10^6^, M**	k_a2_ × 10^−2^, M^−1^s^−1^	k_d2_ × 10^4^, s^−1^
Aβ40					
**0.22 ± 0.12**	7.9 ± 4.4	1.72 ± 0.04	**6.7 ± 4.5**	0.36 ± 0.24	2.38 ± 0.06
Aβ42					
**0.48 ± 0.09**	3.5 ± 0.5	1.66 ± 0.10	**5.6 ± 4.6**	0.28 ± 0.18	1.58 ± 0.26

## Data Availability

Data are contained within the article and Supplementary Materials.

## Supplementary Materials

The following supporting information can be downloaded at: https://www.mdpi.com/article/10.3390/biom15030431/s1, Figure S1. SDS-PAGE analysis of S100A8 (25, 50, and 75 μM), 20 μM Aβ40/Aβ42, and their mixtures cross-linked by 0.02% glutaraldehyde at 37 °C for 1 h (20 mM HEPES-KOH, 140 mM NaCl, 4.9 mM KCl, 2.5 mM CaCl_2_, 1 mM MgCl_2_, pH 7.4). The numbers in the column “M” indicate the molecular masses of markers in kDa. Gel staining by Coomassie Brilliant Blue R-250. Figure S2. The kinetics of HSA–S100A8 interaction examined using BLI (20 mM Tris-HCl, 140 mM NaCl, 4.9 mM KCl, 2.5 mM CaCl_2_, 1 mM MgCl_2_, pH 7.4). Figure S3. Kinetics of Aβ40 (20 μM) fibrillation in the presence of HSA (5 μM), S100A8 (4 μM), S100A9 (4 μM), or a combination of HSA (5 μM) with S100A8 (4 μM) or S100A9 (2 μM) at 30 °C, followed by ThT (10 μM) fluorescence intensity at 485 nm (25 mM Tris-HCl, 140 mM NaCl, 4.9 mM KCl, 2.5 mM CaCl_2_, 1 mM MgCl_2_, pH 7.4 buffer with 0.05% NaN_3_). The standard deviations of the fluorescence signals are indicated. Excitation wavelength of 440 nm. Figure S4. The change in ThT fluorescence in solutions with and without HSA (5 µM) and S100A8 (4 µM)/S100A9 (4 µM) over time. Buffer: 25 mM Tris-HCl, 140 mM NaCl, 4.9 mM KCl, 2.5 mM CaCl_2_, 1 mM MgCl_2_, 0.05% NaN_3_, pH 7.4. Figure S5. Negative staining TEM images of the sample of 4 μM S100A8 (control) incubated without Aβ40. Figure S6. Negative staining TEM images of the sample of 4 μM S100A9 (control) incubated without Aβ40. Figure S7. The change in the ThT fluorescence of mature Aβ fibrils in the absence and presence of S100A8 (4 μM)/S100A9 (4 μM) over time. Buffer: 25 mM Tris-HCl, 140 mM NaCl, 4.9 mM KCl, 2.5 mM CaCl_2_, 1 mM MgCl_2_, 0.05% NaN_3_, pH 7.4. Figure S8. The change in the ThT fluorescence of mature Aβ fibrils (20 μM monomer equivalent) in the absence and presence of HSA (5 μM) over time. The ThT signal did not change significantly and remained stable in the presence of HSA. Buffer: 25 mM Tris-HCl, 140 mM NaCl, 4.9 mM KCl, 2.5 mM CaCl_2_, 1 mM MgCl_2_, 0.05% NaN_3_, pH 7.4. Our findings are in line with the literature [93].

## Appendix A. Evaluation of Distribution of Aβ40 and Aβ42 Between S100A8, S100A9, and HSA

The calculations were carried out according to the following assumptions, scheme of chemical equilibria, and total concentrations of the reagents:

S100A8, S100A9, and HSA interact with Aβ40 and Aβ42 according to the following scheme of chemical equilibria with the equilibrium association constants taken from Table 1 and Table 2:

Aβ40 + S100A8 Aβ40·S100A8			$K_{x}=\frac{\left[A\beta 40\right]\cdot [S100A8]}{\left[A\beta 40\cdot S100A8\right]}=\frac{a\cdot l}{x}=5.1\cdot 10^{-6}\;M$ (Table 1)	$K_{x}=\frac{\left[A\beta 40\cdot S100A8\right]}{\left[A\beta 40\right]\cdot [S100A8]}=\frac{x}{a\cdot l}=2\cdot 10^{5}\;M$ (Table 1)
*a*	*l*	*x*		
Aβ40 + S100A9 Aβ40·S100A9			$K_{y}=\frac{\left[A\beta 40\right]\cdot [S100A9]}{\left[A\beta 40\cdot S100A9\right]}=\frac{a\cdot m}{y}=2.1\cdot 10^{-6}\;M$ (Table 1)	$K_{y}=\frac{\left[A\beta 40\cdot S100A9\right]}{\left[A\beta 40\right]\cdot [S100A9]}=\frac{y}{a\cdot m}=5\cdot 10^{5}\;M$ (Table 1)
*a*	*m*	*y*		
Aβ40 + HSA Aβ40·HSA			$K_{z}=\frac{\left[A\beta 40\right]\cdot [HSA]}{\left[A\beta 40\cdot HSA\right]}=\frac{a\cdot n}{z}=2.2\cdot 10^{-7}M$ (Table 2)	$K_{z}=\frac{\left[A\beta 40\cdot HSA\right]}{\left[A\beta 40\right]\cdot [HSA]}=\frac{z}{a\cdot n}=0.22\cdot 10^{-6}M$ (Table 2)
*a*	*n*	*z*		
Aβ42 + S100A8 Aβ42·S100A8			$K_{p}=\frac{\left[A\beta 42\right]\cdot [S100A8]}{\left[A\beta 42\cdot S100A8\right]}=\frac{b\cdot l}{p}=1.3\cdot 10^{-6}M$ (Table 1)	$K_{p}=\frac{\left[A\beta 42\cdot S100A8\right]}{\left[A\beta 42\right]\cdot [S100A8]}=\frac{p}{b\cdot l}=1.33\cdot 10^{-6}M$ (Table 1)
*b*	*l*	*p*		
Aβ42 + S100A9 Aβ42·S100A9			$K_{q}=\frac{\left[A\beta 42\right]\cdot [S100A9]}{\left[A\beta 42\cdot S100A9\right]}=\frac{b\cdot m}{q}=1.2\cdot 10^{-6}M$ (Table 1)	$K_{q}=\frac{\left[A\beta 42\cdot S100A9\right]}{\left[A\beta 42\right]\cdot [S100A9]}=\frac{q}{b\cdot m}=1.15\cdot 10^{-6}M$ (Table 1)
*b*	*m*	*q*		
Aβ42 + HSA Aβ42·HSA			$K_{r}=\frac{\left[A\beta 42\right]\cdot [HSA]}{\left[A\beta 42\cdot HSA\right]}=\frac{b\cdot n}{r}=4.8\cdot 10^{-7}M$ (Table 2)	$K_{r}=\frac{\left[A\beta 42\cdot HSA\right]}{\left[A\beta 42\right]\cdot [HSA]}=\frac{r}{b\cdot n}=0.48\cdot 10^{-6}M$ (Table 2)
*b*	*n*	*r*		

1. The interactions of Aβ40, Aβ42, S100A8, S100A9, and HSA with other molecules are neglected.
2. The system is in equilibrium, and the equilibrium constants are independent of the other processes in the system.
3. Only the highest equilibrium association constant is taken into consideration for Aβ binding to HSA.
4. The following total concentrations of the proteins were used (the Aβ and HSA concentrations equivalent to those in CSF; the total S100A8 and S100A9 contents corresponding to those in the inflammatory focus):

**Protein**	**Mass Balance**	**Total Concentration, M**	**References**
Aβ40	*a* + *x* + *y* + *z*	1.7·10^−9^	[19,83]
Aβ42	*b* + *p* + *q* + *r*	9·10^−11^	[19,83]
S100A8	*l* + *x* + *p*	9.3·10^−7^	[86]
S100A9	*m* + *y* + *q*	3.8·10^−6^	[86]
HSA	*n* + *z* + *r*	3.0·10^−6^	[89,90]

The system of equations based on the law of mass action and mass balance (see above) was solved numerically using Microsoft Excel 2010 software. The resulting equilibrium molar concentrations of the Aβ-containing components are shown below:

**Component**	**Concentration, M**	**Concentration, M**	**Symbol**
Aβ40	1.0·10^−10^	1.02·10^−10^	*a*
Aβ40·S100A8	1.9·10^−11^	1.88·10^−11^	*x*
Aβ40·S100A9	1.8·10^−10^	1.83·10^−10^	*y*
Aβ40·HSA	1.4·10^−9^	1.39·10^−9^	*z*
Aβ42	8.0·10^−12^	8.00·10^−12^	*b*
Aβ42·S100A8	5.6·10^−12^	5.59·10^−12^	*p*
Aβ42·S100A9	2.6·10^−11^	2.64·10^−11^	*q*
Aβ42·HSA	5.0·10^−11^	5.00·10^−11^	*r*

Thus, about 82% of Aβ40 and 56% of Aβ42 are predicted to be bound to HSA, 12% of Aβ40 and 36% of Aβ42 are bound to S100A8 and S100A9, and 6% of Aβ40 and 9% of Aβ42 are free.
