# Supplementary material for: Binding of Pro-Inflammatory Proteins S100A8 or S100A9 to Amyloid-β Peptide Suppresses Its Fibrillation
Source: Biomolecules. 2025 Mar 17;15(3):431. doi: 10.3390/biom15030431 (PMC11939996; doi:10.3390/biom15030431)
Supplement: Supplementary file 1 [file biomolecules-15-00431-s001.zip › biomolecules-3477502-supplementary.pdf]

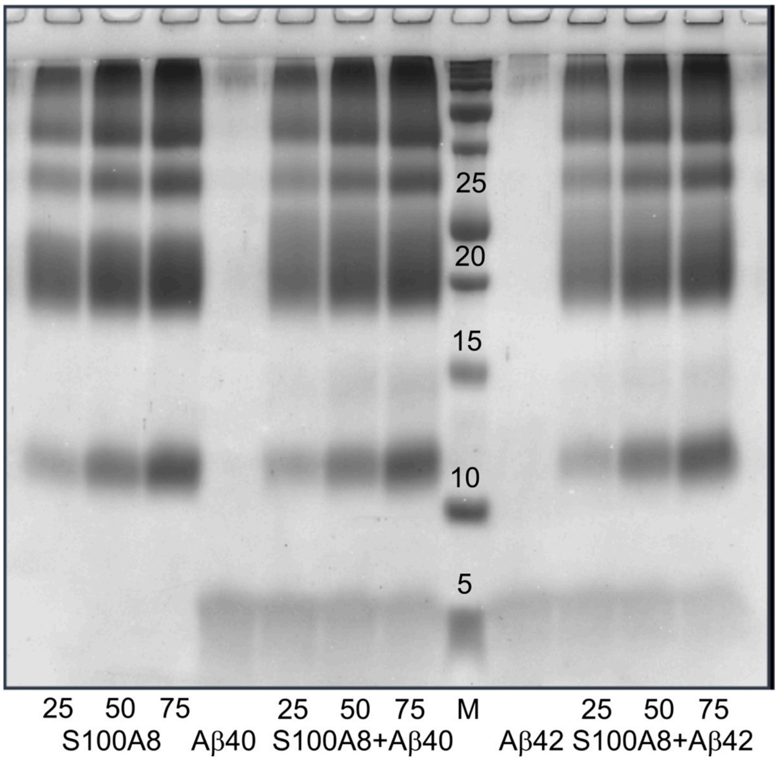

Figure S1. SDS-PAGE analysis of S100A8 (25, 50 and 75 μM), 20 μM Aβ40/Aβ42 and their mixtures cross-linked by 0.02% glutaraldehyde at 37°C for 1 h (20 mM HEPES-KOH, 140 mM NaCl, 4.9 mM KCl, 2.5 mM $CaCl_2$, 1 mM $MgCl_2$, pH 7.4). The numbers in the column "M" indicate molecular masses of markers in kDa. Gel staining by Coomassie Brilliant Blue R-250.

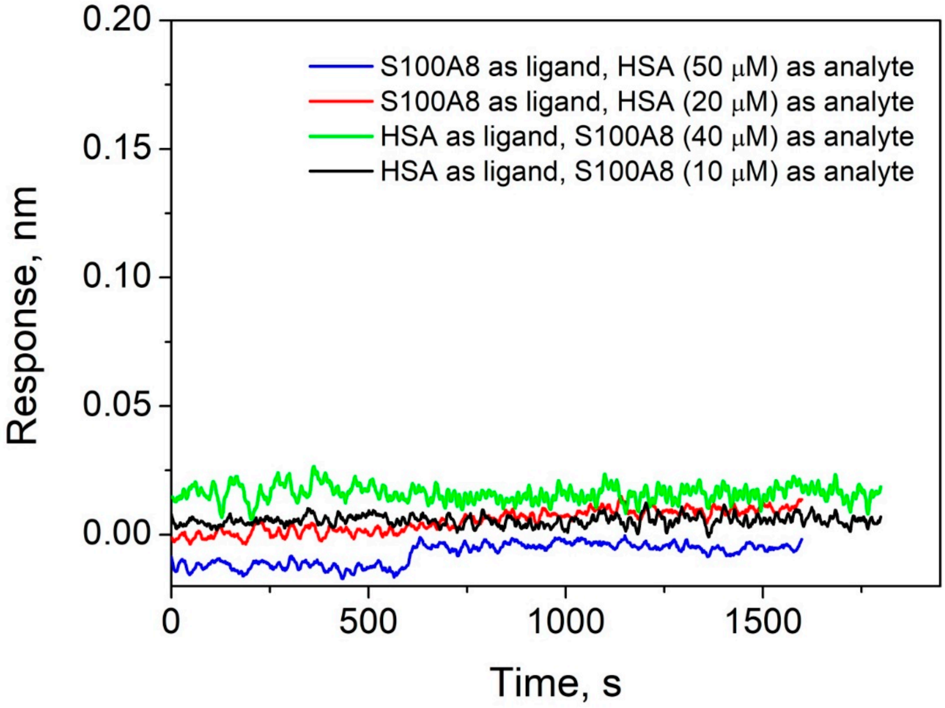

Figure S2. The kinetics of HSA-S100A8 interaction examined using BLI (20 mM Tris-HCl, 140 mM NaCl, 4.9 mM KCl, 2.5 mM $CaCl_2$, 1 mM $MgCl_2$, pH 7.4).

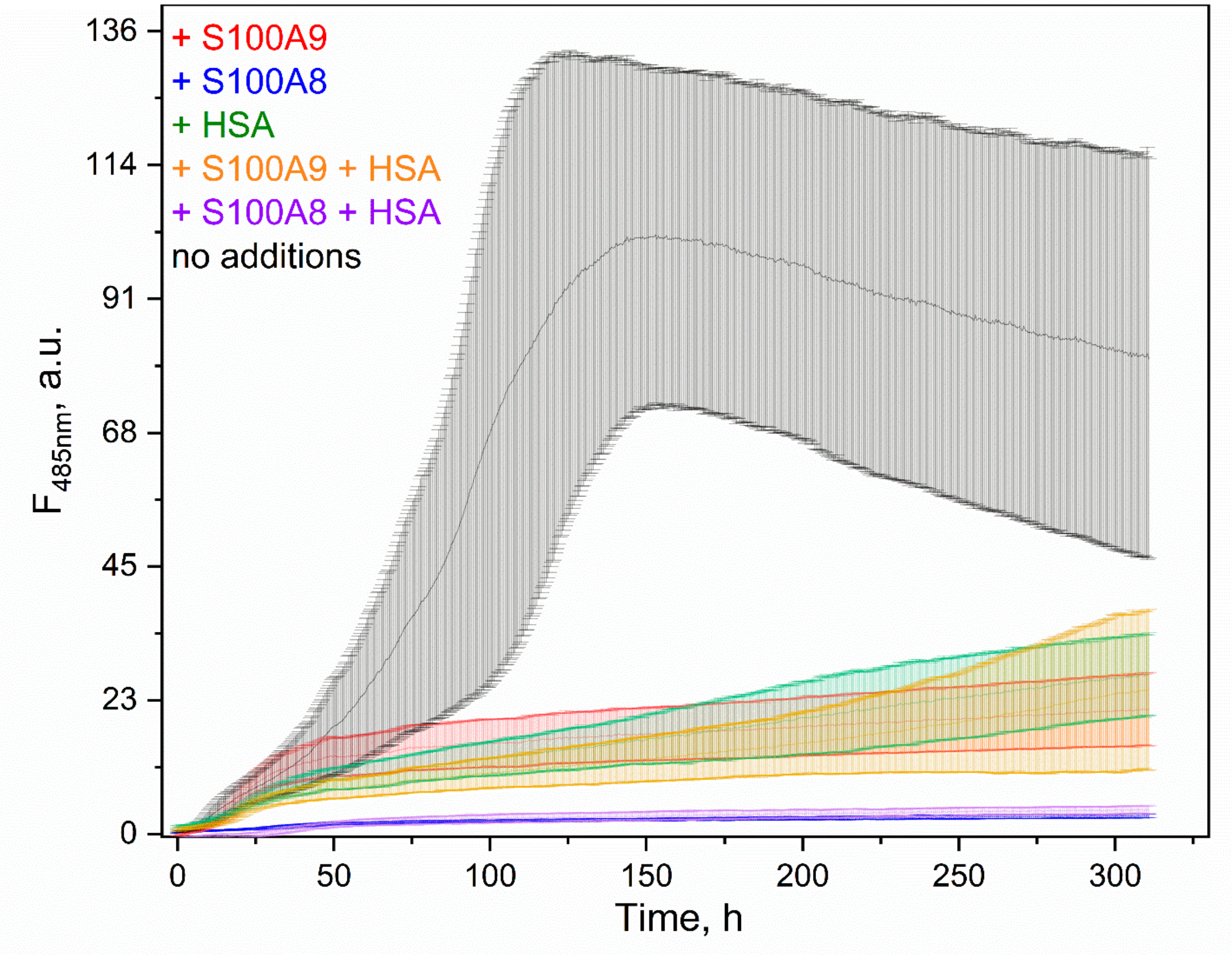

Figure S3. Kinetics of Aβ40 (20 μM) fibrillation in the presence of HSA (5 μM), S100A8 (4 μM), S100A9 (4 μM) or combination of HSA (5 μM) with S100A8 (4 μM) or S100A9 (2 μM) at 30°C, followed by ThT (10 μM) fluorescence intensity at 485 nm (25 mM Tris-HCl, 140 mM NaCl, 4.9 mM KCl, 2.5 mM $CaCl_2$, 1 mM $MgCl_2$, pH 7.4 buffer with 0.05% NaN_3). The standard deviations of the fluorescence signals are indicated. Excitation wavelength of 440 nm.

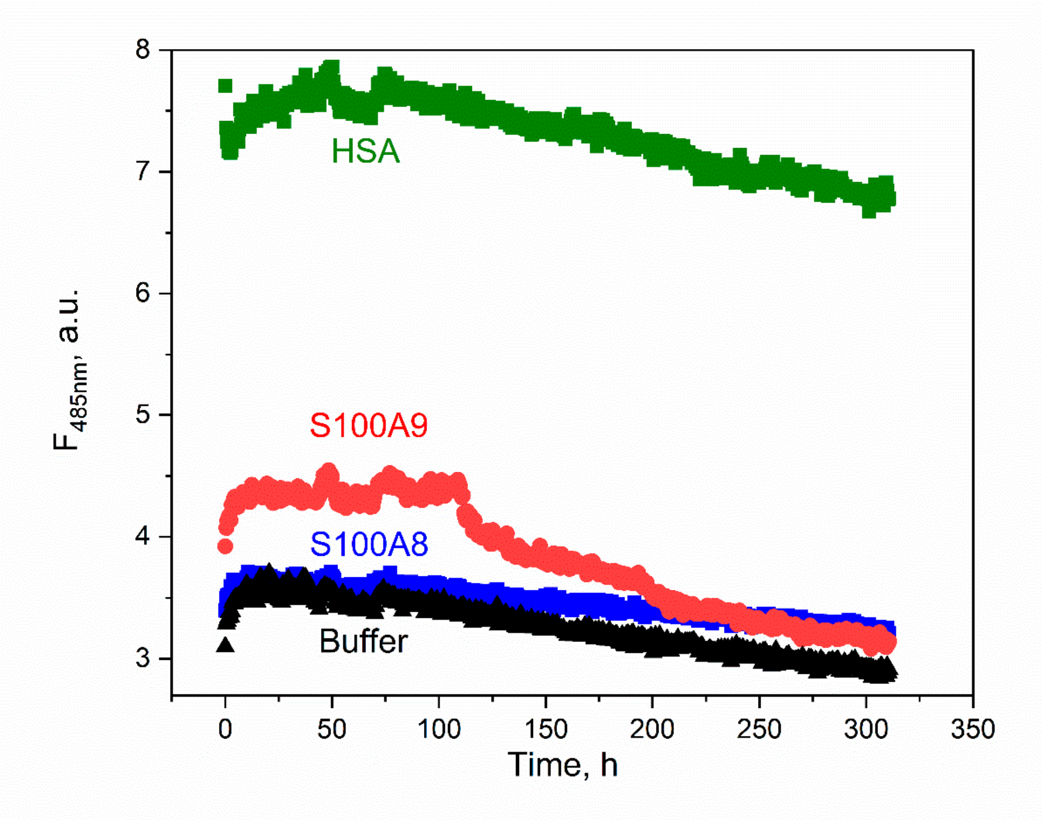

Figure S4. The change in ThT fluorescence in solutions with and without HSA (5 µM), S100A8 (4 µM)/S100A9 (4 µM) over time. Buffer: 25 mM Tris-HCl, 140 mM NaCl, 4.9 mM KCl, 2.5 mM $CaCl_2$, 1 mM $MgCl_2$, 0.05% NaN_3, pH 7.4.

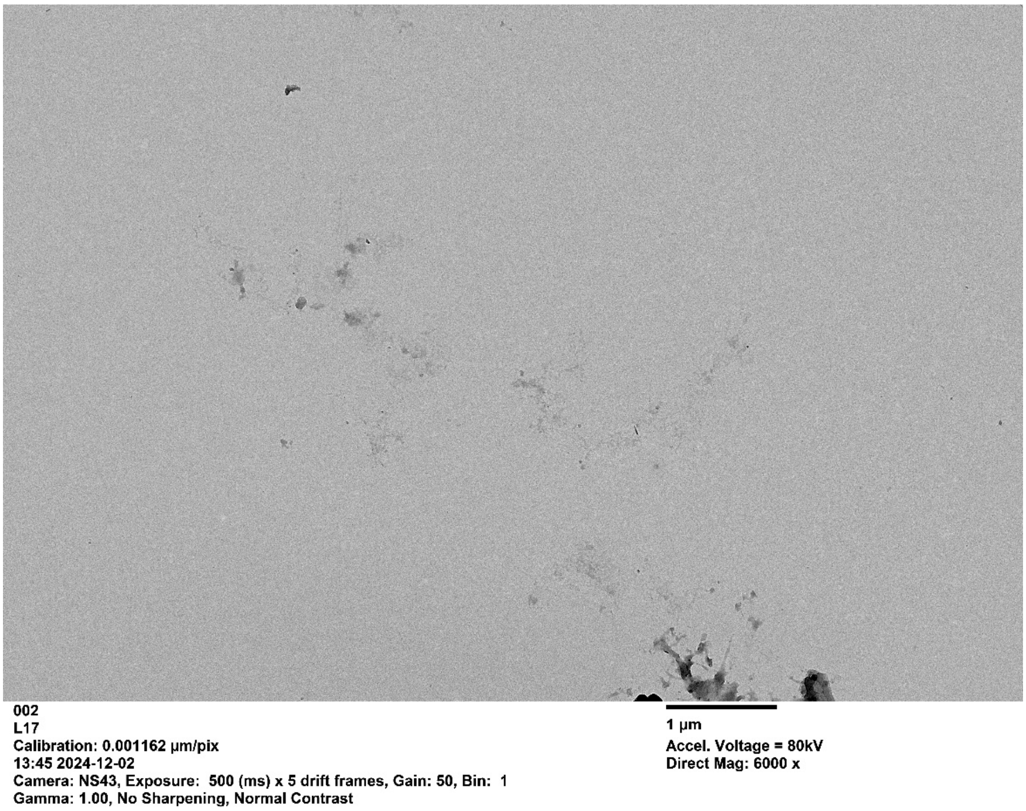

Figure S5. Negative staining TEM images of the S100A8 control sample (4 µM) incubated without Aβ40.

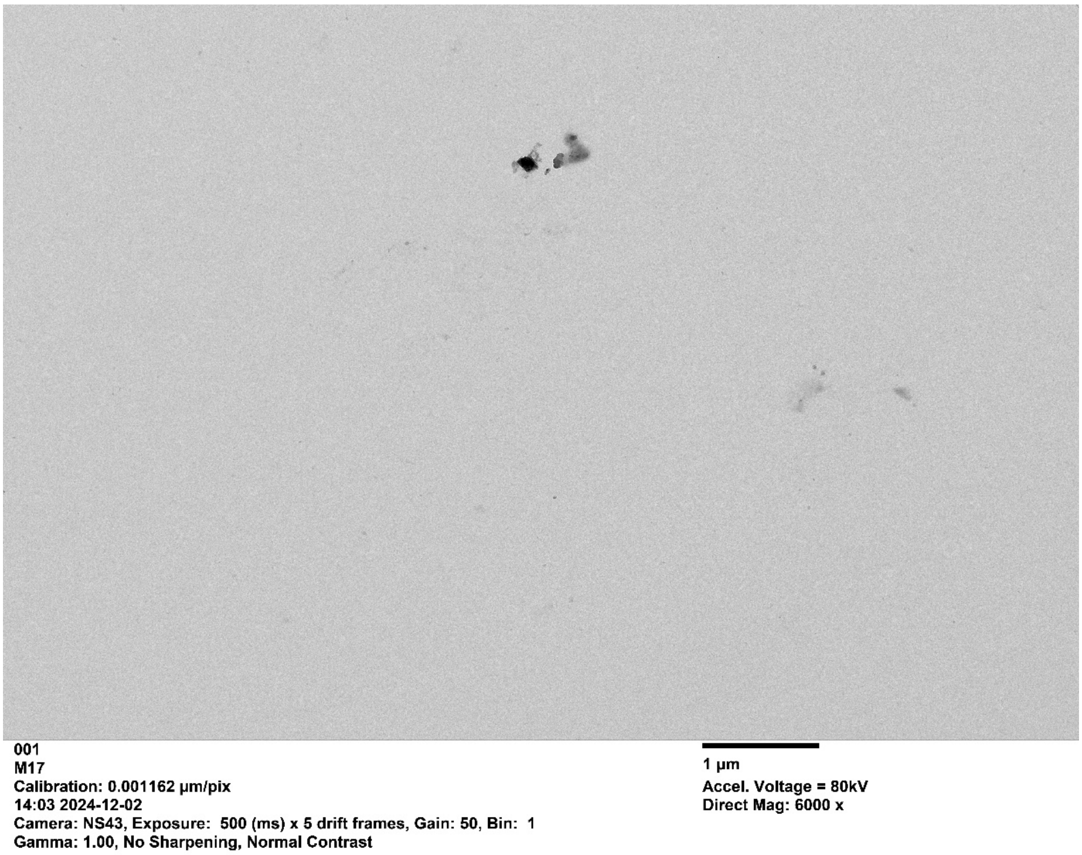

Figure S6. Negative staining TEM images of the S100A9 control sample (4 μM) incubated without Aβ40.

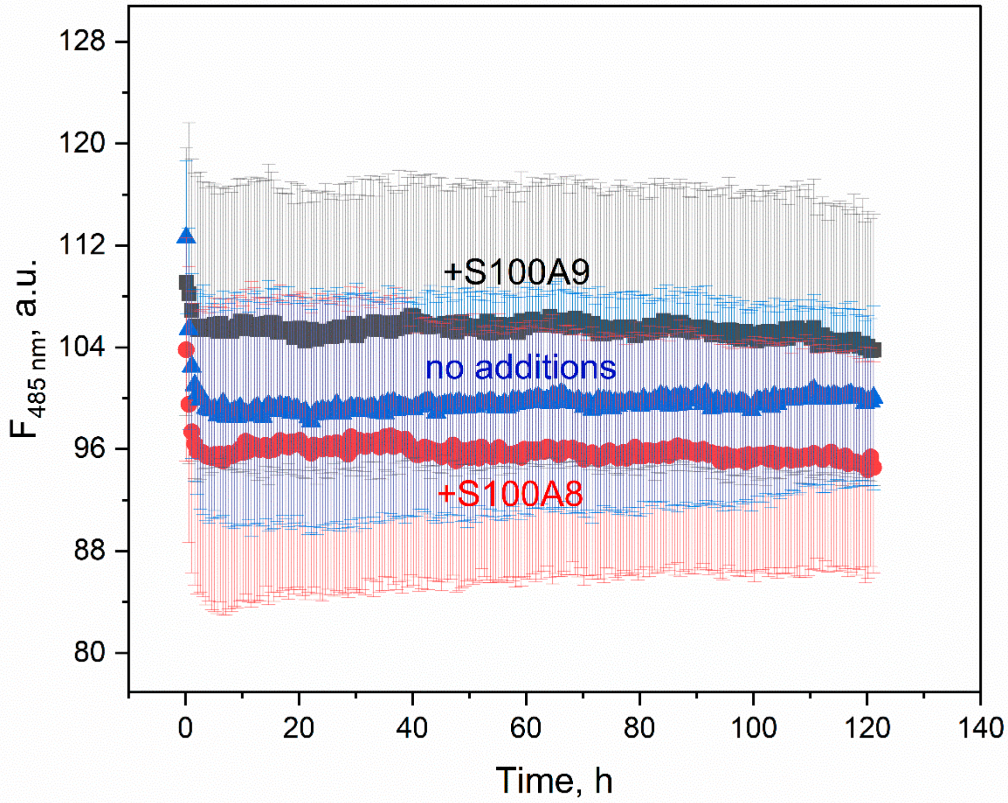

Figure S7. The change in ThT fluorescence of mature Aβ fibrils (20 μM monomer equivalent) in the absence and presence of S100A8 (4 μM)/S100A9 (4 μM) over time. The ThT signal did not alter significantly and remained stable in the presence of S100A8/S100A9. Buffer: 25 mM Tris-HCl, 140 mM NaCl, 4.9 mM KCl, 2.5 mM $CaCl_2$, 1 mM $MgCl_2$, 0.05% NaN_3, pH 7.4.

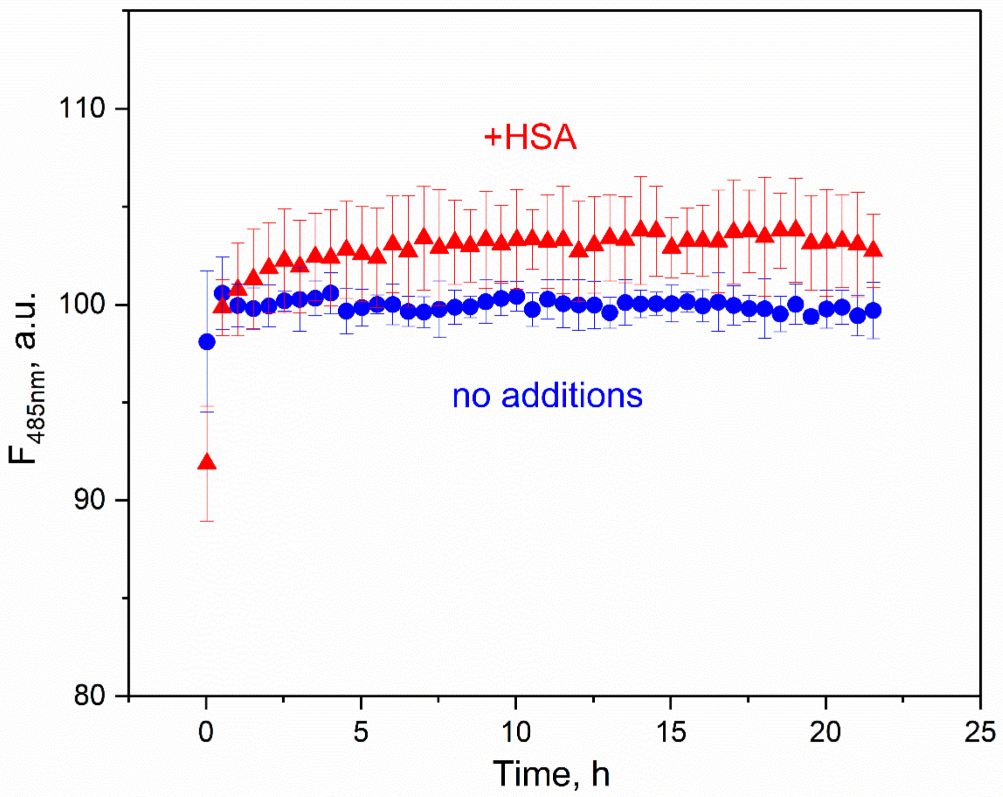

Figure S8. The change in ThT fluorescence of mature Aβ fibrils (20 μM monomer equivalent) in the absence and presence of HSA (5 μM) over time. The ThT signal did not alter significantly and remained stable in the presence of HSA. Buffer: 25 mM Tris-HCl, 140 mM NaCl, 4.9 mM KCl, 2.5 mM $CaCl_2$, 1 mM $MgCl_2$, 0.05% NaN_3, pH 7.4. Our findings are in line with the literature [1].

References:

1. Bode, D.C., et al., *Serum Albumin's Protective Inhibition of Amyloid-β Fiber Formation Is Suppressed by Cholesterol, Fatty Acids and Warfarin.* Journal of Molecular Biology, 2018. **430**(7): p. 919-934.
